# Predicting Recurrent Deficiency and Suboptimal Monitoring of Thiamin Deficiency in Patients with Metabolic and Bariatric Surgery

**DOI:** 10.3390/nu16142226

**Published:** 2024-07-11

**Authors:** Julie M. Parrott, Austen J. Parrott, J. Scott Parrott, Noel N. Williams, Kristoffel R. Dumon

**Affiliations:** 1 Faculty of Health Sciences and Wellbeing, University of Sunderland, Sunderland SR1 3SD, UK; 2 Bariatric Surgery Program, Temple University Hospital, Philadelphia, PA 19140, USA; 3 Behavioral Health, The Child Center of New York, New York, NY 11355, USA; austenparrott@childcenterny.org; 4 School of Health Professions, Rutgers University, Newark, NJ 07102, USA; scott.parrott@rutgers.edu; 5 Division of Gastrointestinal Surgery and Metabolic and Bariatric Surgery, Hospital of the University of Pennsylvania, Philadelphia, PA 19104, USA; noel.williams@pennmedicine.upenn.edu (N.N.W.); kristoffel.dumon@pennmedicine.upenn.edu (K.R.D.)

**Keywords:** vitamin B1 deficiency, thiamine deficiency, micronutrient deficiency, bariatric surgery, Bayesian network, random forest, machine learning

## Abstract

Introduction: Vitamin B1 (thiamine) deficiency (TD) after metabolic and bariatric surgery (MBS) is often insidious and, if unrecognized, can lead to irreversible damage or death. As TD symptoms are vague and overlap with other disorders, we aim to identify predictors of recurrent TD and failure to collect B1 labs. Methods: We analyzed a large sample of data from patients with MBS (*n* = 878) to identify potential predictors of TD risk. We modeled recurrent TD and failure to collect B1 labs using classical statistical and machine learning (ML) techniques. Results: We identified clusters of labs associated with increased risk of recurrent TD: micronutrient deficiencies, abnormal blood indices, malnutrition, and fluctuating electrolyte levels (aIRR range: 1.62–4.68). Additionally, demographic variables associated with lower socioeconomic status were predictive of recurrent TD. ML models predicting characteristics associated with failure to collect B1 labs achieved 75–81% accuracy, indicating that clinicians may fail to match symptoms with the underlying condition. Conclusions: Our analysis suggests that both clinical and social factors can increase the risk of life-threatening TD episodes in some MBS patients. Identifying these indicators can help with diagnosis and treatment.

## 1. Introduction

The risk of thiamin (vitamin B1) deficiency in metabolic and bariatric surgery (MBS) poses a significant threat to patients as well as to programs, hospitals, and clinicians. According to a report by Morton et al. in 2022, 58.1% of complications from MBS could have been prevented, with postoperative care identified as a critical area for improvement (45.1%) [1]. In his review of malpractice closed-claims registry cases (2006 to 2014), 70.9% of treatment events involved delayed, incorrect, or failed treatments [1]. Nutrient deficiencies accounted for only 4.9% of clinical complications and malpractice expenses. However, it is uncertain which cases involved thiamin deficiency (TD) and to what extent nutrient deficiencies were under-reported. Nonetheless, we know that delays or inaccuracies in TD can lead to much worse outcomes for patients and, ultimately, clinicians, programs, and hospitals. For example, in the United States, a client received a substantial monetary settlement of USD 14.1 million due to irreversible neurological damage from Wernicke’s encephalopathy (WE) following routine MBS [2]. Cases of this nature typically involve allegations of care providers failing to adhere to the standard of care in preventing and addressing the early signs of vitamin B1 deficiency [1,3,4,5,6]. 

Alarmingly for clinicians, attorneys are actively advertising and recruiting patients with clearly stated signs and symptoms of TD. Thus, patients with MBS may have a better grasp of TD symptoms than many clinicians. After reviewing cases of TD for possible litigation and publications, there seems to be two key areas where conflicting information has occurred in clinical resources and within daily practice: (1) timing and symptoms of TD, including progression to WE and Korsakoff Syndrome (KS) and (2) criteria used to identify or simply “suspect” TD or WE. Unless a clinician has a clear idea of which symptoms to look for in TD or patients at risk for TD, then in many cases, TD is “out of sight–out of mind”. 

**Figure 1:**
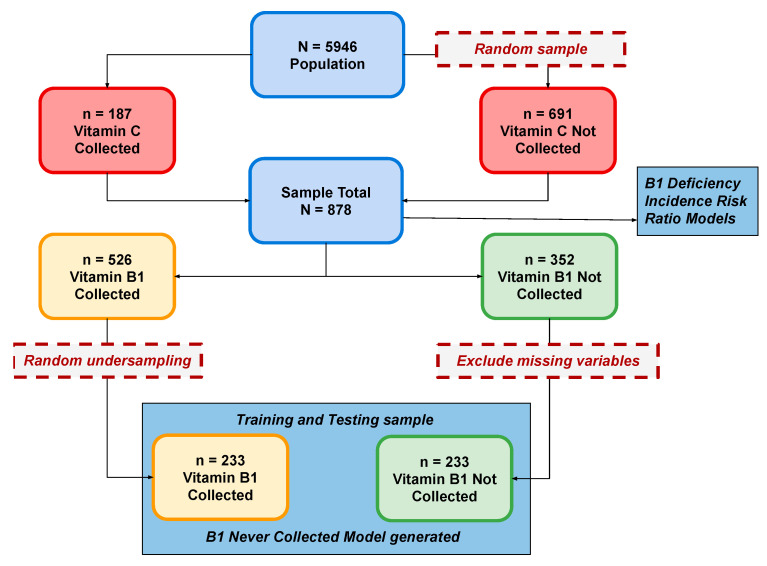
Analysis Flow.

Even with a basic understanding of the clinical presentation of TD, the diagnosis of early-stage TD in patients with MBS may be missed. This could be due to overlapping signs and symptoms between TD and more commonly observed surgical complications [3,7,8,9], as well as alternating symptoms due to changes in health status [10]. The delay or absence of treatment can have serious consequences for patients with MBS, leading to significant delays or missed treatment. If left untreated, TD can cause symptoms to appear rapidly (within two weeks) and quickly progress to WE and KS (Figure 1) [9,11,12].

Understanding which patients with MBS are most at risk for TD is a significant challenge. Even when patients (with alcohol use disorders and patients with AIDS) were known to be at risk for TD, 75–80% of cases with WE confirmed at autopsy were missed [13]. Indeed, all patients with MBS should be considered at some level of risk for TD, and the signs and symptoms of TD should be evaluated regularly. The clinical practice guidelines recommend screening for TD in high-risk patients like females, blacks, and those with specific medical conditions or symptoms. It is crucial to consider the often-overlooked risk factors, as up to 30% of patients with obesity reportedly have TD [14,15]. Furthermore, a recent systematic review by Jawara et al. in 2024 revealed significant racial disparities in the prevalence of TD in laparoscopic gastric bypass and laparoscopic sleeve gastrectomy procedures performed in the U.S. [16]. As the clinical presentation of TD can vary across patients with MBS and can be confounded by other micronutrient deficiencies or common postoperative complaints, it is vital that a set of criteria be developed to identify increased risks of TD and failure to collect vitamin B1 labs in this population. In suspected cases of TD, the recommended treatment is to administer thiamin without waiting for lab results. Even if a patient’s lab results appear normal after treatment, TD could recur if the underlying risk factor is not addressed. Therefore, it is crucial to encourage the patient to seek medical attention and undergo regular checks for TD [14,15]. This prompted our research aims: to determine whether at-risk patient characteristics or lab profiles could predict (1) recurrent TD and (2) failure to collect vitamin B1 labs. 

## 2. Methodology

The sample for this study was adapted from our group’s previous machine learning (ML) study on patients with MBS [17]. Our population consisted of a cohort of adults (>18 years) with MBS seen in a tertiary care center in the US between January 2017 and January 2021. The study was approved by the local institutional review board (IRB #834987) and followed the Transparent Reporting of a Multivariable Prediction Model for Individual Prognosis and Diagnosis (TRIPOD) reporting guidelines.

### 2.1. Sample

From *n* = 5946 patients seen by the program during the study period, all patients (*n* = 187) who were measured for vitamin C (vitC) were included and an additional random sample (*n* = 691) of the remaining patients was generated to create a total sample of *n* = 878 patients (Figure 1).

### 2.2. Measures

Data from 53 common lab measures, as well as demographics and procedure type data, were extracted from the electronic medical record (see Supplementary Materials for a full list of labs). Seven features (variables) were computed for each of the lab measures (ever low, number of times low, ever high, number of times high, lowest abnormal value, highest abnormal value, and lab uncollected), resulting in *n* = 371 lab features available for modeling. We were particularly interested in laboratory tests commonly ordered according to program policies (comprehensive metabolic panel, complete blood count, and nutrient-specific labs). Lab measures from different laboratories were converted to standard units and thresholds for out-of-range lab measures were determined based on laboratory protocols. Additionally, baseline demographics and clinical characteristics, including age, gender, race, body mass index (BMI), marital status, insurance status, MBS procedure type, and number of MBS procedures, were extracted. The primary outcomes of interest were patient characteristics predicted to be associated with (1) recurrent TD and (2) failure to collect vitamin B1 labs.

### 2.3. Data Preparation

For each lab measure, five variables were constructed: ever measured, measure ever abnormal low (the lab value was measured below the reference range at least once in the patient’s record), measure ever abnormal high (the lab value was measured above the reference range at least once in the patient’s record), and number of times abnormal high (number of times the lab value was measured above the reference range) or abnormal low (number of times the lab value was measured below the reference range). For iron deficiency (ID), we constructed three reported measures: (1) Absolute ID with ferritin < 30 (AID), and inflammation-based ID using TSAT% < 20 divided into (2) functional iron deficiency (FID) with ferritin 30–100 (FID30–100), and (3) FID with ferritin > 100 (FID > 100) [18,19,20,21] Because some laboratories did not report actual lab values (e.g., only reporting “out of range” low or high), we could not use these values in the models. The final number of lab variables available was *n* = 265 (53 labs × 5 variables), along with nine demographic and procedure variables.

### 2.4. Statistical Analyses

Descriptive statistics were used to compare thiamin status across demographic and procedure variables. Classical statistical methods, including bivariate and adjusted negative binomial regression models, were used to estimate incident risk ratios (IRRs). Due to the measured overdispersion of recurrent TD, Poisson models were inappropriate. Because the timing and list of labs could not be controlled (different labs measured at different times across patients), reliable multivariable models (i.e., including multiple labs as predictors) could not be created using a traditional statistical approach. *p* < 0.05 was used as the a priori α for significance and the false discovery rate was used to adjust for familywise error [22].

**Figure 2:**
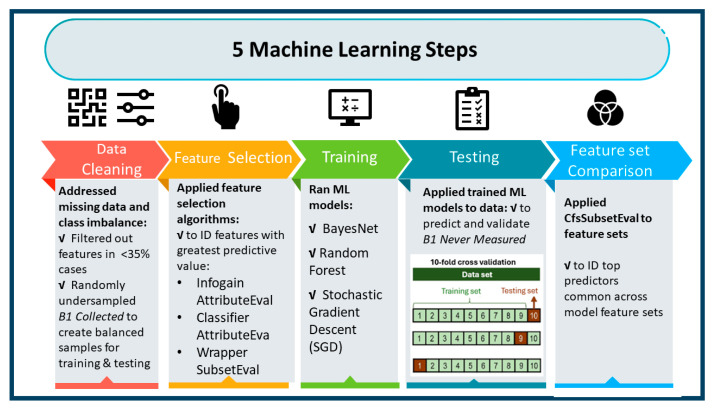
Machine Learning Process.

ML models were used to predict patient characteristics associated with failure to collect vitamin B1 labs (Figure 2). The goal was to create models with high predictive ability as well as identify the most important features across models. Lacking a priori guidance for selecting ML models, we implemented three general types: a Bayesian classifier (Bayesnet [BN]), a decision-tree-based algorithm (Random Forest [RF]), and a function-optimization approach (hinge loss function trained with Stochastic Gradient Descent [SGD]). For data preparation, we randomly undersampled the dominant class (thiamin measured) to create balanced classes [23] and recoded missing values as “uncollected”, dropping features (lab variables) missing in more than 35% of cases. Models were trained and then validated using 10-fold cross-validation [24,25,26] to predict thiamin never measured. For variable selection, cost-efficient algorithms were used to reduce feature spaces to a more manageable 125 variables (these algorithms were *InfoGainAttributeEval* for BN and *ClassifierAttributeEval* for both RF and SGD) and followed up with the more computationally complex *WrapperSubsetEval* method utilizing greedy search to further reduce the variable set optimized to the respective classifier algorithm (BN, RF, SGD). This resulted in three predictive models with top features ranked by importance. The top five variables in all models were then compared. Waikato Environment for Knowledge Analysis (WEKA) (WekaIO, Campbell, CA, USA), a library of Java programs for data pre-processing, classification, regression, clustering, association rules, and visualization, was used for the ML processes and Stata version 17.0 (StataCorp, College Station, TX, USA) was used for classical statistical analyses [27]. 

## 3. Results

Sample characteristics by thiamin status are presented in Table 1. All patient characteristics, except for the number of MBS procedures, were significantly different across thiamin status groups. 

### 3.1. Predictors of Recurrent Thiamin Deficiency

Considering the differences in B1 status based on demographic characteristics, we tested whether demographic characteristics might also be associated with recurrent TD. Three demographic characteristics (Table 2) were associated with an increased risk of recurrent TD: being African American (AA) race, having no domestic partner, and not having private health insurance (e.g., having only Medicare or Medicaid). AA patients had an incidence rate of TD nearly six times (IRR = 5.94, *p* < 0.001) that of White or other race patients.

Because the above three demographic variables were formal confounders, they were considered candidates for adjustment in the lab models. However, given that both African American status and the U.S. system of private/public health insurance are peculiar only to the U.S., we adjusted for the only demographic covariate that had the broadest applicability internationally, viz, no domestic partner. Table 3 presents the vitamin/mineral, blood (RBC) indices, and comprehensive metabolic panel (CMP) labs that were associated with an increased risk of recurrent TD. 

It is notable that the coefficients across lab measures remained relatively consistent across the unadjusted and adjusted models (only low transferrin becoming non-significant in the adjusted model). Many predictors of recurrent TD are highly associated with malnutrition and share overlapping neurological symptoms (e.g., vitamin B6 and folate: aIRR = 4.68 and 3.95, respectively). The fact that several micronutrients were strongly associated with recurrent TD reinforces the Caine revised WE diagnostic criteria using MN deficiencies as one of the modified Wernicke criteria for WE [28].

### 3.2. Failure to Collect Vitamin B1 Labs

All three ML models were successful in predicting thiamin never measured. Across ML models, the AUC was between 76–86%—interpretable as acceptable to excellent discrimination (Table 4).

Out of the top five MN variables of importance to the *ClassifierSubsetEval* algorithm for each predictive model’s feature set, folate was highly important to all three ML models (defined as the number of folds representing the variable), whereas both vitamins B6 and C were in two of the ML models (Table 5). 

This comparison resulted in a list of the most robust predictors of thiamin never measured: abnormal folate (high), abnormal vitamin B6, vitC low, and surgery type. Note, however, that while the type of surgery was an important feature in this analysis, it may not apply to practices with a different mix of procedures.

Table 5 indicates that a range of other labs being ordered predicts thiamin *not* being collected. The implication is that clinicians are seeing symptoms and ordering labs but not connecting the symptoms to TD.

## 4. Discussion

Our findings indicate that recurrent and potentially dangerous TD may be predicted via other commonly obtained labs. Specifically, labs indicating other micronutrient deficiencies, abnormal hematopoietic profile, malnutrition, and fluctuating electrolyte levels may serve as strong signals of a potential underlying TD. This set of TD-signaling labs can help assist differential diagnosis because early-stage TD symptoms are often non-specific and may overlap with complications after MBS [11,12]. A configuration of the other labs that we have identified may be used to flag a potential TD. Because TD can progress from these easily overlooked symptoms to WE/KS, requiring urgent treatment, and in other patients at risk for TD or with already depleted thiamin stores, progression to WE/KS may occur in less than two weeks, it is crucial that clinicians consider the possibility of TD and treat it immediately [11,12]. As noted above, failing to do so can result in catastrophic patient outcomes and even expose physicians and MBS programs to the risk of litigation [1,4,5,6]. These are currently pressing issues, but even more alarming is the fact that as patients age, TD has been identified as a significant risk factor (76.1 RR) for dementia [30] Taylor, 2023.

Our research identified an important aspect of recurrent TD: social determinants. Our findings align with a recent systematic review by Jawara et al., 2024 [16]. In the United States, AA race and public health insurance are highly associated with lower socioeconomic status (SES). This finding is consistent with research highlighting the impact of SES on MBS outcomes [31,32]. Additionally, our research identifies a lack of social support (i.e., no domestic partner) as an additional risk. While this is related to economic challenges (i.e., single income), it may also signal the importance of social support for maintaining micronutrient sufficiency. Thus, in addition to an understanding of the common labs that may signal a TD, clinicians should also be sensitive to race and the economic situation of their patients and how this may be associated with TD.

Additionally, failure to collect vitamin B1 labs may also place patients at risk. In the present study, we developed ML models that differentiated patients who did and who did not have B1 labs collected. The institutional practice at this large MBS program is to provide patients with lab orders, including B1, at their 6-week postoperative clinic visit to be collected and reviewed two weeks prior to their 3-month follow-up. However, for different reasons (e.g., patients transferring to this program from another program, failure to complete lab work, etc.), abnormal thiamin levels can go unnoticed at this early stage, thus missing some patients at higher risk of developing more advanced symptoms of TD. The common features identified across the three models suggest that when symptoms do begin to develop, labs other than thiamin are ordered (e.g., folate, B6, etc.). In short, when thiamin labs are missed as part of standard practice, clinicians do not appear to be connecting the symptoms back to TD.

### 4.1. “Red Flag” Features

As described in Table 3, specific micronutrients have very strong associations with recurrent TD. This is not surprising since it is uncommon to find isolated micronutrient deficiencies and more common to see multiple co-existing micronutrient deficiencies [33,34]. As detailed in Table 3, four clinical patterns may signal a patient is at risk for recurrent TD: (1) deficiency in micronutrients (especially neurotropic vitamins like folate or B6), (2) abnormal blood indices, (3) fluctuating electrolyte balance, and (4) malnutrition indices. Awareness of these associations may help clinicians more quickly suspect TD as the underlying problem. Indeed, these associations can be used to develop decision tools and alerts in the electronic medical record to support clinicians unfamiliar with early TD symptoms.

For some of these clinical patterns, the relation to TD may derive from a shared etiology (e.g., inadequate diet or supplementation regimen), while for others (e.g., blood indices and malnutrition), the association with TD may be the result of thiamin involvement in metabolic pathways. Laboratory signals of malnutrition may be particularly salient since previous research indicates that malnutrition, as suggested by CMP labs, decreases B1 absorption by approximately 70% [8]—suggesting a possible vicious metabolic feedback loop. Nevertheless, the co-occurrence of TD and clinical factors revealed by our models may suggest an underlying physiological mechanism that warrants further research. 

The present study and previous literature describe several possible associations between common micronutrient deficiencies, racial disparities, and TD:

- **Neurotropic vitamins**: Vitamin B1, vitamin B6, and folate act synergistically to maintain a healthy nervous system [35], meaning a deficiency in one may signal a deficiency in the others.
- **Abnormal hematopoietic profile:** Vitamin B6 is needed to help make hemoglobin and without it, anemia can develop [35]. Additionally, ALT is needed for vitamin B6 to function [35]. Thus, B6 deficiency, low ALT, and FID may also indicate a patient at risk for TD.
- **Fluctuating electrolytes:** Electrolytes (calcium, sodium, potassium) vary based on the level of dehydration and repletion, as seen in the present study, which had a high IRR of 1.91–2.87. Thus, patients with fluctuating electrolyte levels, in combination with other micronutrient abnormalities, may be at a greater risk of TD.
- **Malnutrition indices:** a combination of labs from CMP can indicate malnutrition (creatinine, AST/ALT, Total Protein High and Low, Albumin Low, and Glucose Low) and subsequent TD.
- **Racial disparities:** Including race as a variable in research is critical to further explore why there is a disproportionate number of African Americans with TD and other micronutrient deficiencies. Only two out of ten research studies were included in the systematic review by Jawar et. al., 2024 that explored race as a predictor of TD, specifically African Americans as an independent risk factor for TD as reported in sleeve gastrectomy (OR = 3.9, 95% CI 1.25–12.21, *p* = 0.019) [36] and gastric bypass (OR = 6.1, 95% CI 3.0–12.4, P < 0.0001) with race as the only predictor of TD (OR 13.4, 95% CI 5.2–34.5) [37].

Due to differences in the ML algorithms, the list of most important patient characteristics predicting failure to collect B1 differed somewhat (Table 5). Of the top five micronutrients of importance, folate was present in all three ML models, whereas both vitamin B6 and C were in only two of the ML models. Both “folate ever high” and “folate number times measured high” were the top ML predictors of meaningful information. Elevated folate occurs rarely in patients taking too many multivitamins but too much folate can be produced by bacteria in small intestinal bacterial overgrowth (SIBO) with concomitant TD [38]. Previous research suggests at least 40% of patients with MBS have SIBO [15]. 

### 4.2. Criteria for Diagnosis

**Figure 3:**
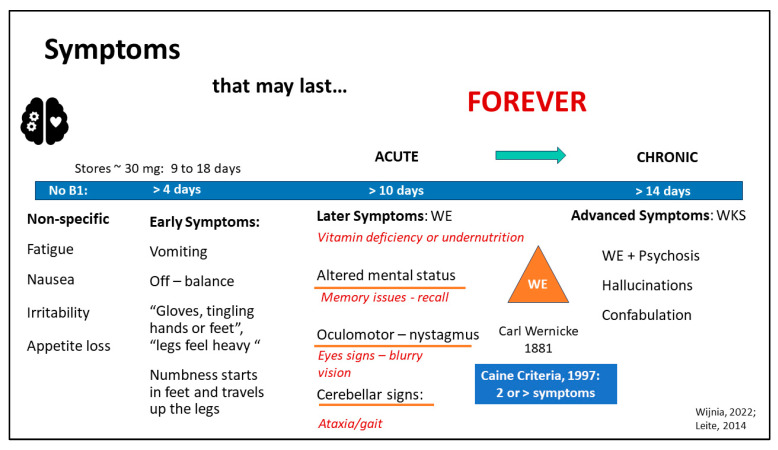
Diagnostic Criteria and Symptoms of Vitamin B1 Deficiency (TD). Approximately: ~, Wernicke’s Encephalopathy: WE, Wernicke’s Korsakoff Syndrome: WKS [11,12,28].

The results of the ML models suggest that clinicians are not making the connection between symptoms and TD. This is, perhaps, unsurprising since accurate identification of TD is complicated by vague early symptoms, such as fatigue, nausea, irritability, and loss of appetite. Storage of vitamin B1 in a patient without obesity or a history of MBS is approximately 30 mg and may last 9–18 days [11]. A condensed list of symptoms of TD, diagnostic criteria, and progression from TD to WE/KS is presented in Figure 3.

Without daily repletion of B1, early symptoms develop, such as vomiting, off-balance or dizziness, and sensations of numbness in the extremities. Complicating timely and accurate diagnosis further, a loss of appetite and vomiting may be both a cause and a complication of TD [11,12]. After about 10 days without B1, later symptoms develop, such as (1) altered mental status, (2) oculomotor signs (nystagmus), or (3) cerebellar signs affecting ataxia or gait [12]. Carl Wernicke identified all three criteria presenting simultaneously as necessary to garner a diagnosis of WE in 1881 [28]. However, in 1997, Caine liberalized the criteria so that altered mental status could include memory issues affecting recall, cerebellar signs included problems with gait and balance, and oculomotor included eye signs, while adding a fourth criterion: dietary deficiency, i.e., vitamin deficiency or undernutrition. Only two or more of these four criteria must be present for WE diagnosis, unlike the prior Wernicke’s criteria, increasing WE diagnostic sensitivity from 31% to 100% [28]. The alignment of these updated criteria with the present study’s findings, including the fact that several micronutrients were strongly associated with recurrent TD, reinforces the revised WE diagnostic criteria using micronutrient deficiencies as one of the modified Wernicke criteria for WE [37]. 

### 4.3. Limitations

The present study was not without limitations. The retrospective study design is an inherent limitation given the dependence on the electronic medical record, as we utilized different sets of labs that were collected from patients at different time points across visits. Had this been a prospective study, individual patients could have been evaluated for symptoms and had dietary intake measured at standard intervals, likely yielding greater accuracy in the prediction of outcomes of interest. However, the utilization of novel ML techniques strengthened this analysis by allowing us to make accurate predictions even with limited data. Moreover, as this sample was drawn from a previous analysis of vitC deficiency, computed ML models may have overestimated the importance of vitC in predicting B1 deficiency. However, given how infrequently vitC is measured, we would have expected this association to be statistically invisible had we taken a random sample of patients who were measured for vitC. Clinically, we would expect water-soluble vitamins with similar storage capacity to vary together. Therefore, the use of vitC as a predictor is not only statistically plausible but also holds practical implications. Despite these limitations, the rigor of the present study was enhanced by robust statistical and predictive modeling techniques.

## 5. Conclusions

Thiamin deficiency is a potentially serious complication of MBS and some patients are at heightened risk. However, it can be difficult to accurately diagnose, which puts patients as well as clinicians and programs at risk. Patterns in commonly collected labs and demographics, in addition to the currently recommended screening in patients at high risk for TD, can be used to help indicate patients who need to be evaluated and monitored more frequently. Awareness of these associations and patients already at high risk of TD may help clinicians more quickly suspect TD as the underlying problem.

## Figures and Tables

**Table 1:** Sample Characteristics.

	B1 Deficient (*n* = 108)		B1 Sufficient (*n* = 418)		B1 Never Measured (*n* = 352)		Total (*n* = 878)		*p*-Value
	*n* or Mean	% or (SD)	*n* or Mean	% or (SD)	*n* or Mean	% or (SD)	*n* or Mean	% or (SD)	
Sex (*n*, %)									
Female	102	94.4	336	80.4	266	75.6	704	80.18	<0.001
Male	6	5.6	82	19.6	86	24.4	174	19.8	
Age (mean, SD)	44.8	11	48.5	12	47.3	13.6	878	12.6	0.021
Ethnicity (*n*, %)									0.019
Not Hispanic/Latino	104	96.3	384	91.9	339	96.3	827	94.2	
Hispanic/Latino	4	3.7	34	8.1	13	3.7	51	5.8	
Race (*n*, %)									<0.001
Black	82	78.9	141	36.2	195	57.4	418	50.1	
White or Other	22	21.1	249	63.9	145	42.7	416	49.9	
Domestic Partner (*n*, %)									0.016
Lives with Partner	31	32.6	182	48.3	112	42	325	44	
Lives Alone	64	67.4	195	51.7	155	58.1	414	56	
Payor (*n*, %)									
Private Insurance or Self	47	43.93	300	72.12	186	54.23	533	61.55	
No Private Insurance	60	56.07	116	27.88	157	45.77	333	38.45	
BMI * (mean, SD)	46.7	9.4	44.1	7.5	46.1	9.6	845	45.1	0.001
Primary Procedure (*n*, %)									<0.001
LSG *	88	82.24	341	81.97	251	71.31	680	77.71	
RYGB *	13	12.15	56	13.46	71	20.17	140	16	
Revision	5	4.67	12	2.88	5	1.42	22	2.51	
Other	1	0.93	7	1.68	25	7.1	33	3.77	
Number of Surgeries (*n*, %)									0.422
One	105	97.22	400	95.69	345	98.01	850	96.81	
Two	2	1.85	15	3.59	6	1.7	23	2.62	
Three or More	1	0.93	3	0.72	1	0.28	5	0.57	

* Body mass index: BMI, Sleeve gastrectomy: LSG, Roux-en-Y gastric bypass: RYGB.

**Table 2:** Demographic Characteristics Associated with Recurrent Thiamin Deficiency.

	IRR *	LCI *	UCI *	*p*-Value
African American	5.94	3.44	10.23	<0.001
No Domestic Partner	2.03	1.26	3.27	0.004
No Private Health Insurance	2.91	1.91	4.45	<0.001

* Incidence risk ratio: IRR; Lower confidence interval: LCI; Upper confidence interval: UCI.

**Table 3:** Lab Measures Associated with Recurrent B1 Deficiency.

	Unadjusted Models				Adjusted Models			
	IRR	LCI	UCI	*p*-Value	IRR	LCI	UCI	*p*-Value
**Vitamins and Minerals**								
Folate Low	4.62	2.22	9.60	<0.001	3.95	1.70	9.19	0.001
Vit B6 Low	4.62	2.96	7.20	<0.001	4.68	3.00	7.30	<0.001
Vit B12 High	2.65	1.73	4.04	<0.001	1.67	1.07	2.58	0.022
Vit C Low	2.65	1.49	4.70	0.001	2.50	1.36	4.59	0.003
Vit D Low	2.82	1.79	4.44	<0.001	2.73	1.71	4.34	<0.001
Calcium Low	2.87	1.89	4.35	<0.001	3.08	1.99	4.76	<0.001
Calcium High	2.24	1.04	4.82	0.040	1.80	0.89	3.66	0.103
Sodium Low	1.91	1.16	3.14	0.011	2.32	1.45	3.73	<0.001
Potassium Low	2.33	1.47	3.70	<0.001	2.07	1.30	3.29	0.002
Potassium High	2.63	1.37	5.04	0.004	2.58	1.40	4.77	0.002
**Blood Indices**								
Ferritin High	4.54	2.75	7.48	<0.001	3.35	2.09	5.36	<0.001
Transferrin Low	1.88	1.04	3.41	0.038	1.62	0.88	2.98	0.118
TSAT Low	2.31	1.18	4.53	0.015	2.55	1.31	4.96	0.006
HCT Low	4.59	2.92	7.19	<0.001	5.58	3.41	9.11	<0.001
HGB Low	4.12	2.59	6.56	<0.001	4.65	2.79	7.75	<0.001
RBC Low	2.17	1.40	3.38	0.001	2.12	1.36	3.31	0.001
AID	2.30	1.45	3.66	<0.001	1.95	1.24	3.08	0.004
FID30–100	3.09	1.58	6.02	0.001	2.97	1.44	6.13	0.003
FID > 100	4.78	3.13	7.30	<0.001	3.63	2.37	5.57	<0.001
**CMP Labs**								
Creatinine High	1.99	1.17	3.38	0.011	2.34	1.31	4.17	0.004
AST Low	3.01	1.97	4.60	<0.001	3.01	1.92	4.71	<0.001
ALT Low	4.54	2.85	7.25	<0.001	4.49	2.70	7.46	<0.001
Total Protein High	3.00	1.79	5.03	<0.001	3.10	1.85	5.19	<0.001
Total Protein Low	1.59	1.01	2.52	0.046	1.79	1.11	2.87	0.017
Albumin Low	2.89	1.89	4.41	<0.001	2.83	1.81	4.42	<0.001
Glucose Low	2.27	1.42	3.64	<0.001	2.27	1.40	3.68	0.001

Absolute iron deficiency with ferritin < 30: (AID), Inflammation-based ID using TSAT% < 20 divided into two functional iron deficiency groups: (FID) with ferritin 30–100 (FID30–100) and FID with ferritin > 100 (FID > 100), Transferrin Saturation%: TSAT, Hematocrit: HCT, Hemoglobin: HGB, red blood cells: RBC, Aspartate aminotransferase: AST, Alanine transaminase: ALT, Comprehensive Metabolic Panel: CMP.

**Table 4:** Comparative Model Performance for Predicting B1 Never Measured.

Algorithm	Number of Features	Accuracy ^a^	AUC ^b^	TD True Positives ^c^	TD False Positives ^d^	TD True Negatives	TD False Negatives
BayesNet	85	75.12%	0.82	0.67	0.17	0.83	0.33
Random Forest	54	80.90%	0.86	0.75	0.13	0.87	025
SGD Hinge Loss	95	75.75%	0.76	0.73	0.22	0.79	0.27

^a^ Accuracy: the proportion of correctly classified observations; ^b^ AUC: Area under the curve; a measure of the improvement in classification ability better than chance. AUC = 1 is perfect prediction, AUC = 0.5 is chance, AUC = 0.7–0.8 “acceptable”, AUC 0.8–0.9 “excellent” [29]; ^c^ True positives: the proportion of TD that were correctly classified as TD; ^d^ False positives: the proportion of patients without TD who were classified as having TD.

**Table 5:** Model Top Variable Set Comparisons (BayesNet, RandomForest, and SGD Hinge Loss).

BayesNet	N Folds	RandomForest	N Folds	SGD Hinge Loss	N Folds
Folate Ever High *	10	Folate Ever High *	10	Folate Times High +	10
Vit B6 Ever High *	10	Vit D Times Low +	10	Vit B6 Times Low **	10
Vit B6 Times Low **	10	HGB Ever Low **	10	Vit C Ever Low *	10
Vit C Ever Low *	10	AST Times High	10	MCV Ever High *	8
Surgery Type **	10	Patient Age	10	Surgery Type **	7

** Variable present in ≥1 fold of both other models, * Variable present in ≥1 fold of one other model, + Lab marker present in ≥1 fold of one other model.

## Data Availability

The datasets presented in this article are not readily available due to a Data Use Agreement administered by the Office of Research Services at Penn Medicine.

## Supplementary Materials

The following supporting information can be downloaded at: https://www.mdpi.com/article/10.3390/nu16142226/s1, List of Features Used for Data Analysis.

## References

[1] Morton J.M., Khoury H., Brethauer S.A., Baker J.W., Sweet W.A., Mattar S., Ponce J., Nguyen N.T., Rosenthal R.J., DeMaria E.J. (2022). First report from the American Society of Metabolic and Bariatric Surgery closed-claims registry: Prevalence, causes, and lessons learned from bariatric surgery medical malpractice claims. Surg. Obes. Relat. Dis..

[2] Arnold Mackles M. (2022). Gastric Bypass Malpractice Yields $14.1 Million Verdict. HealthCare Risk Mangement. https://www.reliasmedia.com/articles/gastric-bypass-malpractice-yields-14-1-million-verdict.

[3] DeMaria E., Trigilio-Black C. (2018). Alarming Increase in Malpractice Claims Related to Wernicke’s Encephalopathy Post Bariatric Surgery: An Alert to Monitor for Thiamine Deficiency. Bariatr. Times.

[4] Tzoumas L., Samara E., Tzoumas K., Tzimas P., Vlachos K., Papadopoulos G. (2021). Medico-Legal Analysis of General Surgery Cases in Greece: A 48 Year Study. Cureus.

[5] Timsit G., Johanet H. (2019). Medico-legal claims in bariatric surgery in France between 2010 and 2015. J. Visc. Surg..

[6] Ratnasingham K., Knight J., Liu M., Karatsai E., Humadi S., Irukulla S. (2017). NHS litigation in bariatric surgery over a ten year period. Int. J. Surg..

[7] Donnino M.W., Vega J., Miller J., Walsh M. (2007). Myths and misconceptions of Wernicke’s encephalopathy: What every emergency physician should know. Ann. Emerg. Med..

[8] Frank L.L. (2015). Thiamin in Clinical Practice. JPEN J. Parenter. Enter. Nutr..

[9] Oudman E., Wijnia J.W., van Dam M., Biter L.U., Postma A. (2018). Preventing Wernicke Encephalopathy After Bariatric Surgery. Obes. Surg..

[10] Smith T.J., Johnson C.R., Koshy R., Hess S.Y., Qureshi U.A., Mynak M.L., Fischer P.R. (2021). Thiamine deficiency disorders: A clinical perspective. Ann. N. Y. Acad. Sci..

[11] Leite H.P., de Lima L.F.P. (2014). Thiamine (Vitamin B1) Deficiency in Intensive Care: Physiology, Risk Factors, Diagnosis, and Treatment. Diet and Nutrition in Critical Care.

[12] Wijnia J.W. (2022). A Clinician’s View of Wernicke-Korsakoff Syndrome. J. Clin. Med..

[13] Sechi G., Serra A. (2007). Wernicke’s encephalopathy: New clinical settings and recent advances in diagnosis and management. Lancet Neurol..

[14] Parrott J., Frank L., Rabena R., Craggs-Dino L., Isom K.A., Greiman L. (2017). American Society for Metabolic and Bariatric Surgery Integrated Health Nutritional Guidelines for the Surgical Weight Loss Patient 2016 Update: Micronutrients. Surg. Obes. Relat. Dis..

[15] Mechanick J.I., Apovian C., Brethauer S., Timothy Garvey W., Joffe A.M., Kim J., Kushner R.F., Lindquist R., Pessah-Pollack R., Seger J. (2020). Clinical Practice Guidelines for the Perioperative Nutrition, Metabolic, and Nonsurgical Support of Patients Undergoing Bariatric Procedures—2019 Update: Cosponsored by American Association of Clinical Endocrinologists/American College of Endocrinology, The Obesity Society, American Society for Metabolic and Bariatric Surgery, Obesity Medicine Association, and American Society of Anesthesiologists. Obesity.

[16] Jawara D., Ufearo D.M., Murtha J.A., Fayanju O.M., Gannon B.M., Ravelli M.N., Funk L.M. (2024). Racial disparities in selected micronutrient deficiencies after bariatric surgery: A systematic review. Surg. Obes. Relat. Dis..

[17] Parrott J.M., Parrott A.J., Rouhi A.D., Parrott J.S., Dumon K.R. (2023). What We Are Missing: Using Machine Learning Models to Predict Vitamin C Deficiency in Patients with Metabolic and Bariatric Surgery. Obes. Surg..

[18] Camaschella C., Girelli D. (2020). The changing landscape of iron deficiency. Mol. Asp. Med..

[19] Cappellini M.D., Comin-Colet J., de Francisco A., Dignass A., Doehner W., Lam C.S., Macdougall I.C., Rogler G., Camaschella C., Kadir R. (2017). Iron deficiency across chronic inflammatory conditions: International expert opinion on definition, diagnosis, and management. Am. J. Hematol..

[20] Cappellini M.D., Musallam K.M., Taher A.T. (2020). Iron deficiency anaemia revisited. J. Intern. Med..

[21] Benotti P.N., Wood G.C., Dove J.T., Kaberi-Otarod J., Still C.D., Gerhard G.S., Bistrian B.R. (2021). Iron deficiency is highly prevalent among candidates for metabolic surgery and may affect perioperative outcomes. Surg. Obes. Relat. Dis..

[22] Green G.H., Diggle P.J. (2007). On the operational characteristics of the Benjamini and Hochberg False Discovery Rate procedure. Stat. Appl. Genet. Mol. Biol..

[23] Galar M., Fernandez A., Barrenechea E., Bustince H., Herrera F. (2011). A review on ensembles for the class imbalance problem: Bagging-, boosting-, and hybrid-based approaches. IEEE Trans. Syst. Man Cybern. Part C (Appl. Rev.).

[24] Cooper G.F., Herskovits E. (1992). A Bayesian method for the induction of probabilistic networks from data. Mach. Learn..

[25] Breiman L. (2001). Random forests. Mach. Learn..

[26] Rodriguez J.D., Perez A., Lozano J.A. (2009). Sensitivity analysis of k-fold cross validation in prediction error estimation. IEEE Trans. Pattern Anal. Mach. Intell..

[27] Witten I.H., Frank E. (2002). Data mining: Practical machine learning tools and techniques with Java implementations. ACM Sigmod Rec..

[28] Caine D., Halliday G., Kril J., Harper C. (1997). Operational criteria for the classification of chronic alcoholics: Identification of Wernicke’s encephalopathy. J. Neurol. Neurosurg. Psychiatry.

[29] Hosmer D.W., Lemeshow S., Sturdivant R.X. (2013). Applied Logistic Regression.

[30] Taylor R.A., Gilson A., Chi L., Haimovich A.D., Crawford A., Brandt C., Magidson P., Lai J.M., Levin S., Mecca A.P. (2023). Dementia risk analysis using temporal event modeling on a large real-world dataset. Sci. Rep..

[31] Johnson L.P., Asigbee F.M., Crowell R., Negrini A. (2018). Pre-surgical, surgical and post-surgical experiences of weight loss surgery patients: A closer look at social determinants of health. Clin. Obes..

[32] Khalid S.I., Maasarani S., Shanker R.M., Becerra A.Z., Omotosho P., Torquati A. (2022). Social determinants of health and their impact on rates of postoperative complications among patients undergoing vertical sleeve gastrectomy. Surgery.

[33] Schiavo L., Scalera G., Pilone V., De Sena G., Capuozzo V., Barbarisi A. (2015). Micronutrient defi ciencies in patients candidate for bariatric surgery: A prospective, preoperative trial of screening, diagnosis, and treatment. Int. J. Vitam. Nutr. Res..

[34] Berger M.M., Shenkin A., Schweinlin A., Amrein K., Augsburger M., Biesalski H.K., Bischoff S.C., Casaer M.P., Gundogan K., Lepp H.L. (2022). ESPEN micronutrient guideline. Clin. Nutr..

[35] Calderón-Ospina C.A., Nava-Mesa M.O. (2020). B Vitamins in the nervous system: Current knowledge of the biochemical modes of action and synergies of thiamine, pyridoxine, and cobalamin. CNS Neurosci. Ther..

[36] Tang L., Alsulaim H.A., Canner J.K., Prokopowicz G.P., Steele K.E. (2018). Prevalence and predictors of postoperative thiamine deficiency after vertical sleeve gastrectomy. Surg. Obes. Relat. Dis..

[37] Clements R.H., Katasani V.G., Palepu R., Leeth R.R., Leath T.D., Roy B.P., Vickers S.M. (2006). Incidence of vitamin deficiency after laparoscopic Roux-en-Y gastric bypass in a university hospital setting. Am. Surg..

[38] Bushyhead D., Quigley E.M.M. (2022). Small Intestinal Bacterial Overgrowth-Pathophysiology and Its Implications for Definition and Management. Gastroenterology.

