# Supplementary material for: Predicting Recurrent Deficiency and Suboptimal Monitoring of Thiamin Deficiency in Patients with Metabolic and Bariatric Surgery
Source: Nutrients. 2024 Jul 11;16(14):2226. doi: 10.3390/nu16142226 (PMC11280029; doi:10.3390/nu16142226)
Supplement: Supplementary file 1 [file nutrients-16-02226-s001.zip › nutrients-3100150-supplementary.pdf]

## List of Features Used for Data Analysis

| Unique MEASURE_LABEL |
|---|
| A1C |
| ALBUMIN |
| ALBUMIN_GLOBULIN_RATIO |
| ALKPHOS |
| ALT |
| AST |
| BILI_DIR |
| BILIT |
| BUN_CREATININE |
| CALCIUM |
| COPPER |
| CREATININE |
| CRP |
| D2 |
| D3 |
| FERRITIN |
| FOLATE |
| GFR_AA |
| GFR_NONAA |
| GFR_UNKNOWN_RACE |
| GLUCOSE |
| HCT |
| HGB |
| H. pylori |
| HS_CRP |
| IPTH |
| IRON |
| IRON_SATURATION |
| MAGNESIUM |
| MCHC |
| MCV |
| MMA |
| PHOSPHATE |
| POTASSIUM |
| PROTEIN_TOTAL |
| RBC |
| RDW |
| RET_HE |

| |
|---|
| SODIUM |
| TIBC |
| TRANSFERRIN |
| TSAT_P |
| TSH |
| VITA |
| VITA_RETINOL |
| VITA_RETINYL_PALMITATE |
| VITB1 |
| VITB12 |
| VITB6 |
| VITC |
| VITD |
| WBC |
| ZINC |
